# Development of Antifungal Peptides against Cryptococcus neoformans; Leveraging Knowledge about the *cdc50Δ* Mutant Susceptibility for Lead Compound Development

**DOI:** 10.1128/spectrum.00439-22

**Published:** 2022-04-04

**Authors:** Robert J. Tancer, Yina Wang, Siddhi Pawar, Chaoyang Xue, Gregory R. Wiedman

**Affiliations:** a Department of Chemistry and Biochemistry, Seton Hall University, South Orange, New Jersey, USA; b Public Health Research Institute, Department of Microbiology, Biochemistry and Molecular Genetics, New Jersey Medical School, Rutgers University, Newark, New Jersey, USA; University of Guelph

**Keywords:** Cryptococcus neoformans, antimicrobial agents, antimicrobial peptides, drug synergy, fungal meningitis, fungi, immunocompromised hosts, lead compounds, mutants, resistance

## Abstract

Cryptococcus neoformans is a major fungal pathogen that often causes life-threatening meningitis in immunocompromised populations. This yeast pathogen is highly resistant to the echinocandin drug caspofungin. Previous studies showed that Cryptococcus lipid translocase (flippase) is required for the caspofungin resistance of that fungus. Mutants with a deleted subunit of lipid flippase, Cdc50, showed increased sensitivity to caspofungin. Here we designed an antifungal peptide targeting the P4-ATPase function. We synthesized stable peptides based on the Cdc50 loop region to identify peptides that can sensitize caspofungin by blocking flippase function and found that myristylated peptides based on the “AS15 sequence” was effective at high concentrations. A modified peptide, “AW9-Ma” showed a MIC of 64 μg/mL against H99 wild type and a fractional inhibitory concentration (FIC) index value of 0.5 when used in combination with caspofungin. Most notably, in the presence of the AW9-Ma peptide, C. neoformans wild type was highly sensitive to caspofungin with a MIC of 4 μg/mL, the same as the *cdc50*Δ mutant. Further assays with flow cytometry showed inhibition of the lipid flippase enzyme activity and significant accumulation of phosphatidylserine on the cell membrane surface. Using a fluorescently labeled peptide, we confirmed that the peptide co-localized with mCherry-tagged P4-ATPase protein Apt1 in C. neoformans. Structure-activity relationship studies of the AW9 sequence showed that two lysine residues on the peptide are likely responsible for the interaction with the P4-ATPase, hence critical for its antifungal activity.

**IMPORTANCE** The authors have developed a lead compound peptide antifungal drug targeting a protein from the organism Cryptococcus neoformans. Binding of the drug to the target fungal protein causes charged lipid molecules to be retained on the surface. This peptide works in synergy with the existing antifungal drug caspofungin. Echinocandin drugs like caspofungin are one of the few classes of existing antifungals. Due to the high concentrations needed, caspofungin is rarely used to treat C. neoformans infections. The authors believe that their new compound provides a way to lower the concentration of caspofungin needed to treat such infections, thus opening the possibility for greater utility of these antifungal.

## INTRODUCTION

Cryptococcus neoformans is a problematic caspofungin resistant fungus which is the most concerning for immunocompromised patients such as people with HIV/AIDS, those undergoing cancer chemotherapy or transplantation. C. neoformans is the leading cause of fungal meningitis in HIV/AIDS patients and is responsible for ∼15% AIDS related deaths each year (1). The treatment options for fungal infections are limited to only three available drug classes: triazoles, polyenes (amphotericin B), and echinocandins. Of these classes, triazoles are fungistatic and amphotericin B is fungicidal yet highly toxic. Echinocandins are the newest fungicidal drug class with fewer side effects against a variety of invasive fungal infections. These drugs are inactive against Cryptococcus and therefore not a treatment option for patients with such a challenging prognosis (2, 3) Thus, there is an urgent need to identify novel drug targets and to develop new antifungals. Our goal was to develop a drug that would allow for targeted, increased C. neoformans sensitivity to echinocandin drug caspofungin. The *de novo* design of effective new drugs is exceedingly expensive and challenging. A more reasonable approach is to examine the resistance mechanisms of microbes and ameliorate the resistance with rationally designed drugs that counteract the resistance mechanism, potentiating the drug’s effectiveness.

Echinocandins are a class of drugs that inhibit the (1, 3)-β-D glucan synthase, a ubiquitous fungal enzyme that is responsible for polymerizing the carbohydrate component of the fungal cell wall (4). Inhibiting this process leads to apoptosis of cells. The understanding of Cryptococcus resistance mechanism to echinocandins has been incremental. It was proposed that one or more of three possibilities are responsible: (i)the target itself is resistant; (ii) caspofungin is excluded from cells; or (iii) caspofungin is degraded. Maligie and Slitrennikoff were able to show, in an optimized *in vitro* assay, that the Cryptococcal (1, 3)-β-D glucan synthase was inhibited by echinocandins. Their work suggested that the enzyme itself is not likely responsible for the apparent drug resistance and, additionally, that caspofungin is not rapidly degraded by C. neoformans (5). Thus, resistance is most likely due to an exclusion mechanism. Therefore, our goal was to examine a possible mechanism addressing exclusion as a route to increase caspofungin sensitivity in C. neoformans.

Recently, mutation studies discovered an essential role of Cryptococcus lipid flippase in echinocandin resistance (6). The lipid flippase is composed of the core P4 type ATPase (P4-ATPase) and a regulatory subunit Cdc50 (7). The same study found that fungal mutants with a *CDC50* gene of the 250 amino acid exocytoplasmic loop region were highly sensitive to caspofungin. This loop region of Cdc50 has been reported in other organisms to bind to the P4-ATPase to regulate flippase activity (8). The MIC value of caspofungin in the wild-type strain H99 was decreased in *cdc50*Δ from 16 μg/mL to 4 μg/mL. The P4-ATPase flippase is responsible for translocating exoplasmic phosphatidylserine (PS) to the intracellular side of the cell membrane. Within these mutants, an increase in cell-surface PS was found to correlate to an increase in echinocandin sensitivity in broth microdilution MIC assays. We therefore hypothesized that peptides that could interfere with the P4-ATPase activity of C. neoformans would increase the sensitivity to caspofungin. Such peptides would prove to be a novel combinational therapeutic agent together with echinocandins like caspofungin.

In this study, we identified a peptide in the loop region of the Cdc50 protein for which an anti-Cdc50 antibody bound strongly to the protein. We generated a stable peptide based on this sequence by adding a lipid tail. Antifungal activity tests showed that a nine amino acid peptide significantly increased the fungicidal activity of caspofungin in C. neoformans. Peptide treatment leads to increased surface PS exposure. Our results demonstrated that this peptide could serve as a lead candidate for a potential peptide-based inhibitors of fungal lipid flippase function and for future antifungal development.

## RESULTS AND DISCUSSION

### Peptide design.

**FIG 1:**
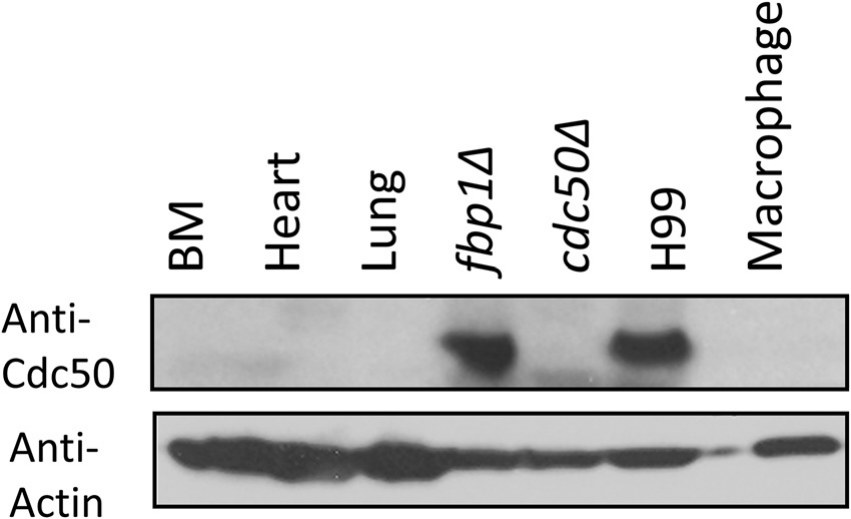
Western blot analysis of a Cdc50 antibody. Antibodies were tested for its binding to total proteins from animal tissues (BM, Heart and Lung), Cryptococcus neoformans cells (H99, *fbp1Δ*, *cdc50Δ*), and murine macrophages in SDS-PAGE. The signal detected by an Anti-actin antibody was used as a protein loading control.

**FIG 2:**
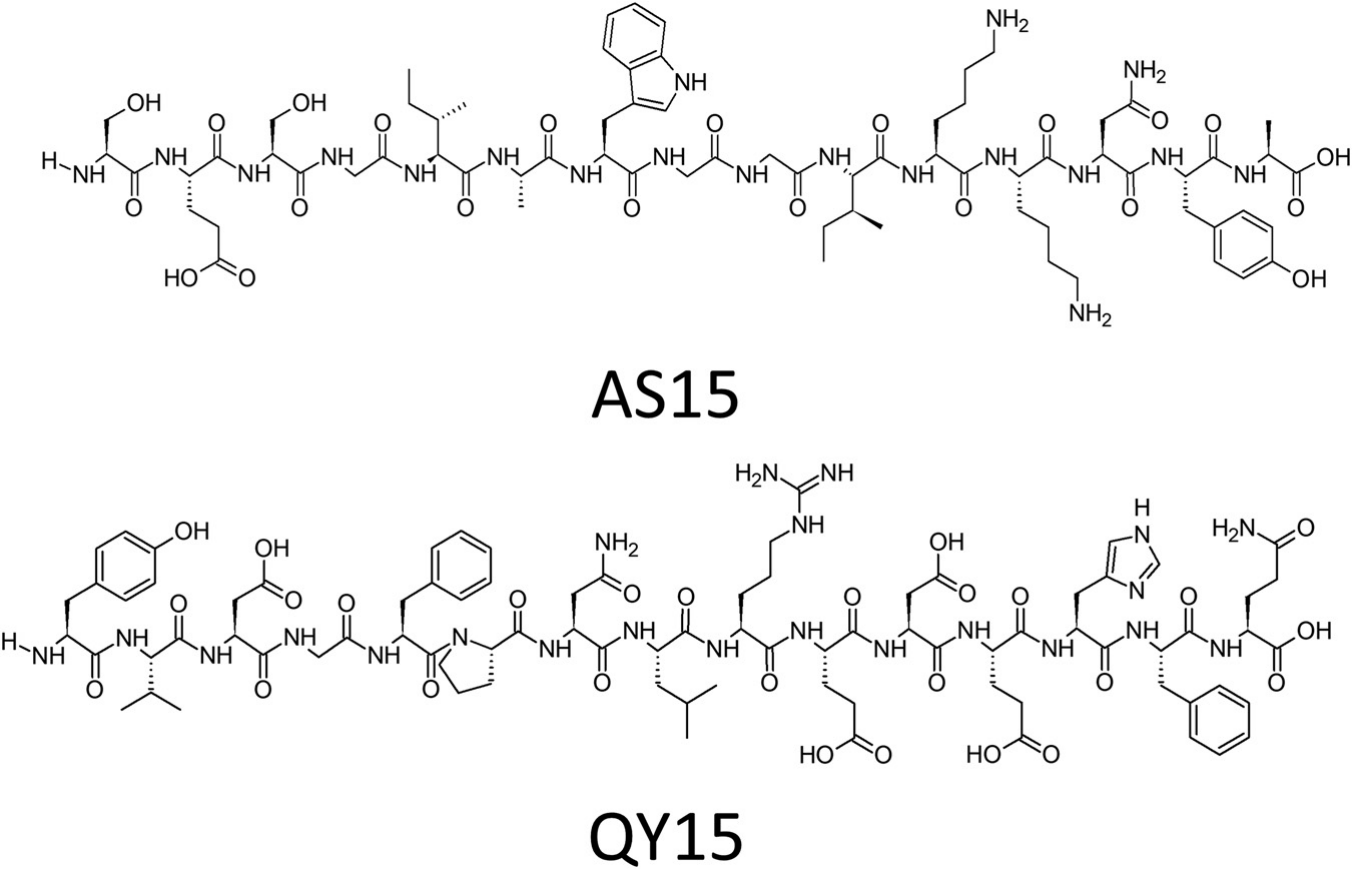
Structure of the peptides AS15 and QY15. These sequences were selected because of their antigenic properties in the development of Anti-cdc50 antibodies. AS15-dreived antibodies were found to be active against C. neoformans protein isolates whereas QY15-derived antibodies were not active (data not shown).

We designed a peptide drug based on a portion of the outer loop region from the Cdc50 protein. The C. neoformans capsules and cell wall are thick multicomponent systems, that provide a barrier preventing large molecules from binding to its target (9). Peptide drugs are low molecular weight molecules that are potentially less excluded from the cell than larger antibody fragments (10). We selected regions of the Cdc50 outer loop that were confirmed to be exposed in H99 spheroplasts using anti-Cdc50 antibodies and identified one polyclonal antibody that showed positive signal in a Western blot assay. This AS15 peptide-based antibody was shown to target total protein extracts from H99 cells while also not labeling the *cdc50*Δ mutant total protein extract nor animal cells or macrophages (Fig. 1). The binding regions (AS15 and QY15) for two antibodies (one positive and one negative in Western blot) were shown below and are part of the fungal Cdc50 loop (Fig. 2). We hypothesized that peptides based on these sequences could disrupt the Cdc50 interaction with P4-ATPase to block the flippase function, hence making C. neoformans susceptible to caspofungin, similar to the *cdc50Δ* mutant.

In our own previous work we found that myristic acid-modified peptides are able to work synergistically with other antimicrobial drugs (11). We hypothesized that a lipid tail would be essential for interaction with a lipid-flipping enzyme. We synthesized and characterized both lipidated and non-lipidated versions of these AS15 and QY15 peptides. Such flippase inhibitors could slow the growth of C. neoformans, and also improve the activity of caspofungin (12). Given the apparent importance of the flippase activity for caspofungin sensitivity, we created and tested a series of peptides with different fatty acid tail (FAT) lengths to determine the ideal length for activity. Then a series of myristylated peptides with reduced amino acid counts were made to determine which section of the peptide was most important for flippase binding (Table 1).

**TABLE 1:** Sequences of antifungal peptides studied^*a*^

N’ lipidation	Name^*b*^	Sequence	Mass	N’ lipidation	Name^*b*^	Sequence	Mass
None	AS15	SESGIAWGGIKKNYA	1,580.76	Decanoic C10	AS15-Da	SESGIAWGGIKKNYA	1,733.94
Ma C14	AS15-Ma	SESGIAWGGIKKNYA	1,791.13	Palmitic C16	AS15-Pa	SESGIAWGGIKKNYA	1,819.18
None	QY15	YVDGFPNLREDEHFQ	1,865.98	Ma C14	AW9-Ma	WGGIKKNYA	1,246.56
Ma C14	QY15-Ma	YVDGFPNLREDEHFQ	2,076.34	Ma C14	KS9-Ma	SGIAWGGIK	1,098.40
Acetic C2	AS15-Ac	SESGIAWGGIKKNYA	1,622.80	Ma C14	GS9-Ma	SESGIAWGG	1,072.27
Hexanoic C6	AS15-Ha	SESGIAWGGIKKNYA	1,678.91	Palmitic C16	AW9-Pa	WGGIKKNYA	1,274.62

a Ac, acetic acid; Ha, hexanoic acid; Da, decanoic acid; Ma, myristic acid; and Pa, palmitic acid.

b Each peptide name is displayed along with any possible C terminal modifications.

### Lipidated loop region peptides show MIC toward C. neoformans.

We examined each peptide for the activity against C. neoformans wild-type strain H99 in both the presence and the absence of caspofungin. The initial set of peptides (QY15, QY15-Ma, AS15, AS15-Ma) were comprised of the AS15 and QY15 fungal loop region sequences and the lipidated versions of both. These peptides were termed “Group 1”. The nonlipidated peptides from the first group were unable to inhibit the growth of the H99 strain at any concentration up to 128 μg/mL (Table 2). Similarly, the fractional inhibitory concentration (FIC) index for such peptides was 1 or greater, indicating little to no improvement of caspofungin activity or even antagonistic effects. Visual inspection of the MIC plates suggested that stronger and more confluent growth in the wells dosed with high concentration of un-lipidated AS15 peptide compared to the no drug control. These peptides, contrary to inhibiting the growth of C. neoformans, may be involved in growth enhancement as a nutrient supplement.

**TABLE 2:** FIC index values of caspofungin with original loop peptides against C. neoformans^*a*^

Peptide	FIC index
QY15	>1
QY15-Ma	>1
AS15	>1
AS15-Ma	0.504

a FIC index values are shown. The following are commonly accepted standard interpretations of FIC Index (FICI) values: 2 > FIC > 1 antagonistic, FICI = 1 autonomous, 1 > FICI > 0.5 additive, FICI ≤ 0.5 drug synergy.

Unlike the nonlipidated peptides, the lipidated versions showed promising antifungal activity against C. neoformans. These peptides exhibited an MIC value of greater than 128 μg/mL, however growth was significantly reduced compared to the no-drug control. Thus, these peptides may not provide any benefits to growth as observed with the nonlipidated peptides. We observed an apparent additive effect when combining AS15-Ma and caspofungin in checkerboard assays against H99. In the presence of 0.5 μg/mL AS15-Ma, the MIC of caspofungin was reduced from 16 μg/mL to 8 μg/mL against H99. An accurate MIC for many of these peptides alone could not be established due to limitations in solubility. For those peptides that did cause the MIC of caspofungin to decrease, an FIC index value was calculated assuming a conservative MIC of 128 μg/mL (see Equation 1). This suggested that the FIC index for such peptides as 0.504, a value between 0.5 and 1 and an additive effect. Thus, even a small amount of the myristylated peptide was able to improve the effectiveness of caspofungin against the WT strain. Similar results were seen with the QY-15-Ma peptide. Unlike QY15-Ma, however, AS15-Ma exhibited an MIC value of 8 μg/mL could be determined against the *cdc50Δ* mutant strain (Table S1 in the supplemental material). This suggested that AS15-Ma was the more potent peptide.

### Fatty acid tail length is important for activity.

We selected AS15-Ma as a lead molecule, due to the increased activity against *cdc50*Δ (Supplemental Material). We sought to determine if the fatty acid tail length was crucial for antifungal activity and drug synergy. Fatty acid tail lengths between acetic (C2) and decanoic (C10) were conjugated to the N-terminus of the AS15 peptide using their respective anhydrides. We also synthesized a palmitic acid version of the AS15 peptide (AS15-Pa). These peptides comprised our second group (Group 2) of possible antifungals and or caspofungin potentiators. As seen in Table 3, none of the FAT scan peptides with tails smaller than C14 inhibited the growth of C. neoformans. The effects were autonomous or mildly antagonistic. The palmitic labeled AS15 showed a similar, additive effect compared to the Group 1 peptide (0.5 < FIC Index <1). These results suggest a minimum fatty acid tail length is required for peptide activity. Additionally, this shows that increased hydrophobicity from the longer tail lengths does not lead to inhibition or complications for delivery through the cell capsule.

**TABLE 3:** FIC index values of caspofungin with peptides of varying fatty acid tail lengths against C. neoformans^*a*^

Peptide	FIC index
AS15-Pa	0.504
AS15-Ma	0.504
AS15-Da	>1
AS15-Ha	>1
AS15-Aa	>1

a FIC index values are shown. The following are commonly accepted standard interpretations of FIC Index (FICI) values: 2>FIC > 1 antagonistic, FICI = 1 autonomous, 1>FICI > 0.5 additive, FICI ≤ 0.5 drug synergy.

### Truncation of the peptide region shows improved activity.

The initial peptide length of 15 amino acids was chosen based on previous data using loop-targeting antibodies. We sought to determine if truncation of the peptides to regions shorter than 15 amino would alter activity. We sought to retain the tryptophan W7 due to the fact that it can be utilized in a variety of important bioanalytical and biophysical applications (13–15). We also hypothesized that the important amino acids in AS15 were the lysines: K11 and K12 due to lysine’s known importance of charged amino acids for antimicrobials (16, 17). For these reasons we synthesized a third group (Group 3) of truncated peptides which contained both lysines (AW9), one lysine (KS9), or lacking both lysines (GS9). We found that removal of either lysine decreased the activity of the Group 3 peptides and removed their ability to sensitize C. neoformans toward caspofungin (Table 4). We further found that AW9-Ma exhibited improved activity, having a detectable MIC value of 64 μg/mL against C. neoformans WT as well as an FIC Index of 0.5. We note that an FIC Index value of ≤0.5 denotes drug synergy (18). We also would note the FIC of caspofungin is 4 μg/mL, which is the value obtained in literature for the MIC of the *cdc50*Δ against caspofungin. A small amount of group 3 palmitic labeled peptide, 0.5 μg/mL, did improve the effectiveness of caspofungin against the H99, lowering the MIC from 16 to 8 μg/mL. This suggests that the myristic peptide tail is optimal for the activity of our peptides.

**TABLE 4:** FIC index values of caspofungin with differentially truncated peptides acid tail lengths against C. neoformans^*a*^

Peptide	FIC index
AW9-Ma	0.5
AW9-Pa	0.75
KS9-Ma	>1
GS9-Ma	>1

a FIC index values are shown. The following are commonly accepted standard interpretations of FIC Index (FICI) values: 2>FIC > 1 antagonistic, FICI = 1 autonomous, 1>FICI > 0.5 additive, FICI ≤ 0.5 drug synergy.

### Peptides showed minimal hemolytic activity.

**FIG 3:**
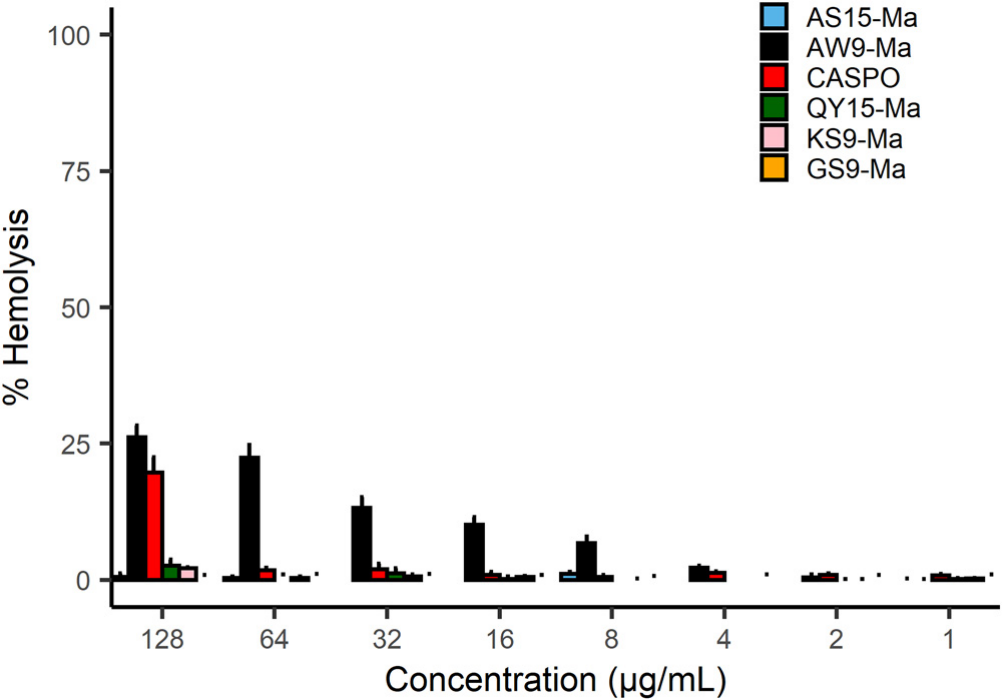
Hemolysis caused by proteins and caspofungin (Caspo) at varying concentrations in μg/mL. Each sample tested against human RBC at 5 X 10^8^ cells/mL. Fractional lysis was determined with respect to a control of 1 μL of Triton X-100 added to control wells.

Human red blood cells (RBCs) are known to contain a mammalian version of P4-ATPase (19) These flippases contribute to their membrane integrity (19–21). We therefore aimed to determine if our peptides could contribute to cell death or hemolysis of RBCs in a similar mechanism compared to C. neoformans. Method development for the hemolysis assay was carried out to ensure the measurements were within the linear response range of the instrument. Different concentrations of RBC’s and supernatant transfer volumes were analyzed (Fig. S2 in the supplemental material). The working number of RBCs used was selected based on the range of cell concentrations seen in patient samples and used in the literature (22). All peptides showed minimal hemolytic activity, directly proportional to its concentration (Fig. 3). Of the peptides tested, AW9-Ma did show a concentrate-dependent minimal amount of hemolysis. This would be consistent with interference of the P4-ATPase and possible damage of the RBC membrane. We note, however, that at the concentrations needed for synergy, AW9-Ma causes low amounts of hemolysis. Additionally, this amount of RBC hemolysis is lower than what is observed for known cytotoxic antimicrobial peptides (23).

### Peptide activity correlates with increased surface phosphatidylserine.

**FIG 4:**
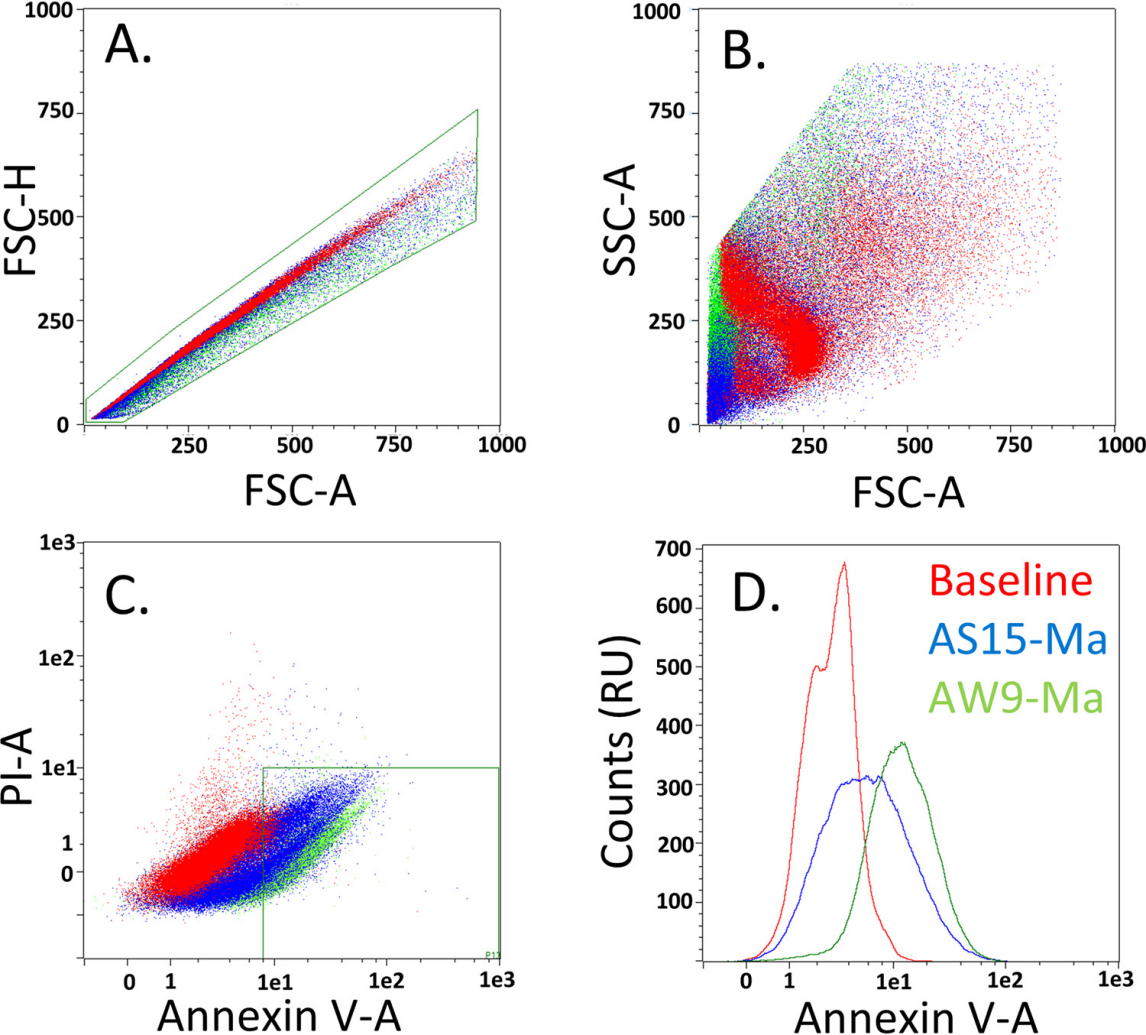
Flow cytometry data of C. neoformans exposed to peptides AS15-Ma and AW9-Ma. Cells were gaited first to ensure only singlets were collected (A) and then for morphology (B) Data were collected and gaited for signal as indicated (C). Finally, the distribution of counts (in relative units, RU) in that region was determined (D).

We sought to determine whether the activities of the peptides were related to the lipid-flipping activity of the P4-ATPase. For this reason, we conducted flow cytometry experiments using AW9-Ma or AS15-Ma, propidium iodine, and Alexa Fluor 350 labeled annexin V (AF350-annexin V). The intensity of the AF350-annexin V signal corresponds to concentration of PS on the exofacial side of the membrane. The propidium iodine signal corresponds to cell viability. We found that, compared to a no drug control, there is a significant increase in AF350-annexin V signal, and hence exofacial PS on C. neoformans cells caused by the peptides (Fig. 4). Single cells were first selected and gated based on morphology to ensure homogeneity among the cell population (4A, 4B). We examined cells with low propidium iodine intensity (PI-A < 9 RU) and high AF350-annexin V intensity (Annexin V-A > 9 RU) (4C, 4D). Under these gating parameters there should be, respectively, low nonspecific cell death and high exofacial PS. For the control sample, the percentage of cells found within this range was only 0.2 ± 0.1% while for the AS15-Ma treated cells the amount was 12.1 ± 2.0% and AW9-Ma treated cells the amount was 67.6% ± 2.3%. These values were found to be mutually statistically significantly different with *P*-values < 0.05 in two-sample Student’s t-tests. This suggests that, while the peptides were capable of increasing exofacial PS via disruption of P4-ATPase activity, they alone were not permeating the cell membrane. Additionally, increased peptide activity among those peptides tested correlated with increased exofacial PS.

### Peptide AW9-Ma co-localizes with a P4-ATPase Apt1 on cells.

**FIG 5:**
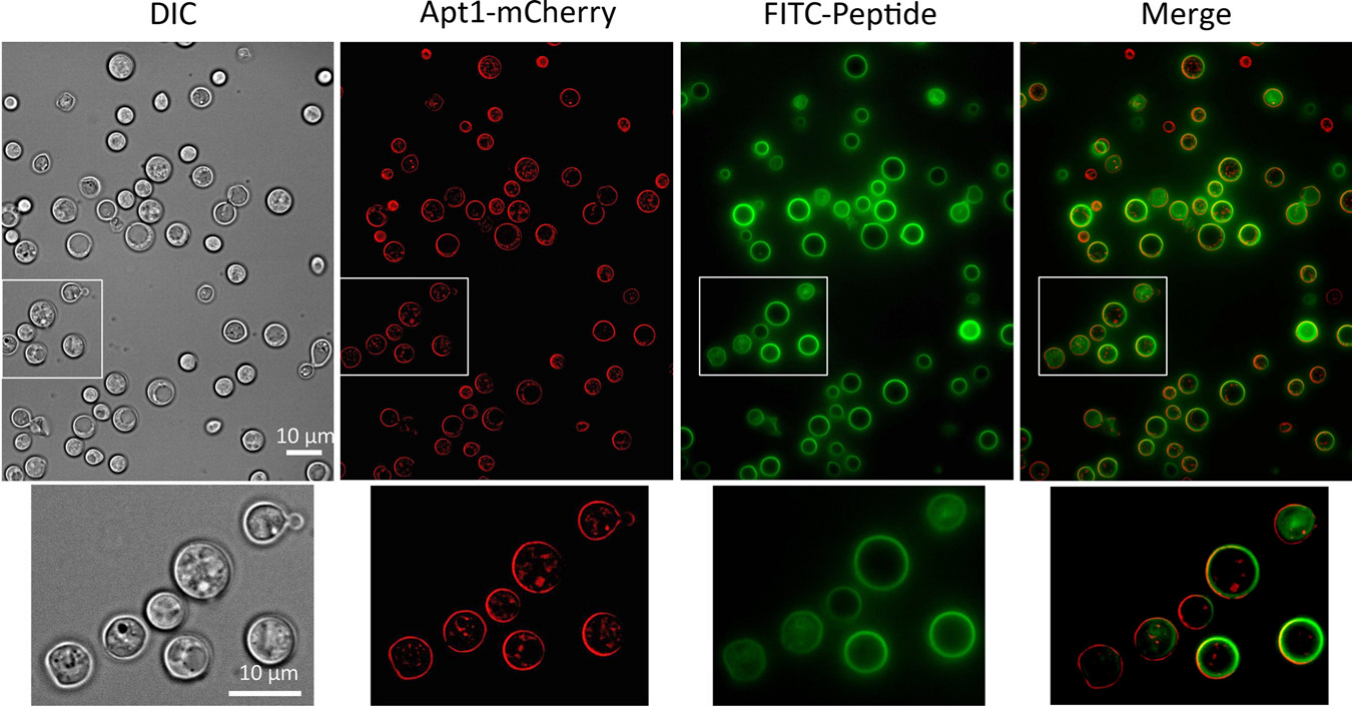
Imaging of FITC-AW9-Ma peptide co-localization with Apt1:mCherry. The FITC labeled AW9-Ma peptide was added to the fungal strain expressing Apt1:mCherry fusion protein for 10 min before observed under fluorescence microscope. The insert area of the images was highlighted at the bottom of each image. Bar, 10 μm.

A FITC fluorescently labeled version of the AW9-Ma peptide (FITC-AW9-Ma) was generated to visualize the interaction of the peptides with the cells. We did this to determine whether the peptide was interacting with the surface of the fungi, the location of the P4-ATPase, or some internal portion of the cell. Along the same lines, we also generated a C. neoformans strain CUX281 that expresses an mCherry tagged P4-ATPase Apt1 (Apt1:mCherry). Co-localization of the signal would indicate a possible interaction between the peptide and the enzyme. This fungal strain was co-incubated with FITC-AW9-Ma peptide for 10 min and observed under fluorescence microscope. We found that the FITC signal was mostly colocalized with the mCherry signal (Fig. 5). These images show a lack of signal, and hence lack of peptide, in the internal portions of the cells. Rather, peptide accumulated either on the surface or was retained within the cell capsule. This again suggests that the peptides are active on the cell surface rather than an internal target and are not cell penetrating.

### Wild-type H99 treated with peptides exhibits cdc50Δ-like SDS susceptibility phenotype.

**FIG 6:**
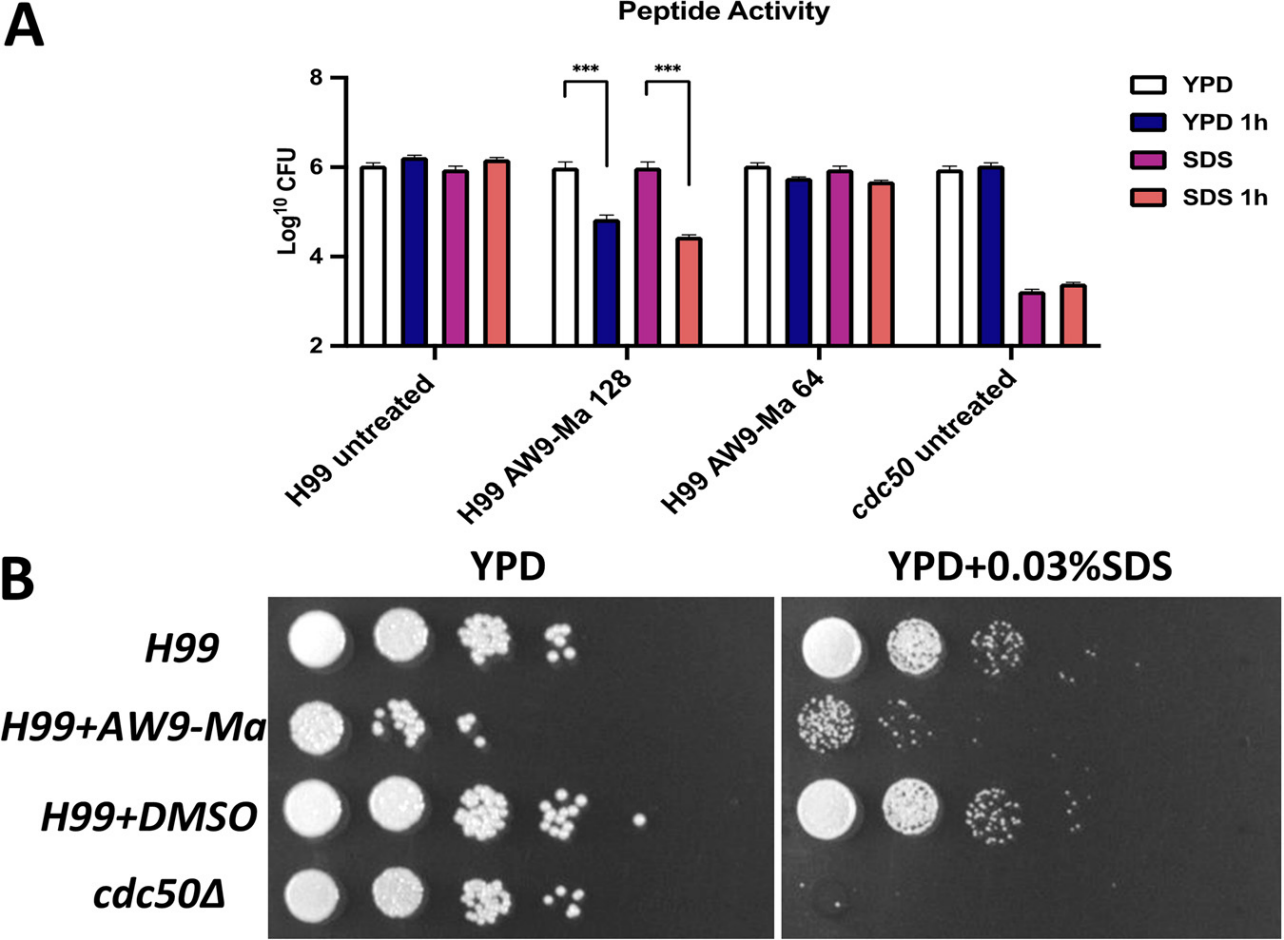
Sensitivity of peptide-treated fungal cells to SDS. Relative CFU are shown above. Growth of the indicated strains was conducted in YPD media and SDS-modified media (A). Colony dilution assays were conducted for strains with the associated treatment in YPD or YPD + 0.03% SDS (B). The error bars indicate standard deviations for three experiments. ***, *P* < 0.001 (determined by ordinary one-way ANOVA).

The C. neoformans mutant strain *cdc50*Δ exhibits increased sensitivity to sodium dodecyl sulfate (SDS) due to a membrane integrity defect (6) We aimed to determine if this phenotype can be seen in H99 cells treated with the most potent peptide, AW9-Ma. Briefly, cultures of C. neoformans were grown in YPD and then spotted on YPD plates containing 0.03% SDS. To test the effect of peptide AW9-Ma on cell phenotype, yeast cells were inoculated into liquid YPD with 128 μg/mL or 64 μg/mL peptide and co-incubated for 1h at 30°C (Fig. 6A). Plate spotting assays were performed with the fungi alone (H99, *cdc50*), with peptide (H99+Aw9-Ma) or with DMSO control (H99+D). Indeed, C. neoformans cells pretreated with AW9-Ma at 128 μg/mL for 1 h showed a significantly reduced growth in terms of CFU on agar medium with 0.03% SDS compared to untreated or DMSO controls (Fig. 6B). These data indicate that AW9-Ma treatment of C. neoformans wild type cells phenocopies the *cdc50*Δ mutant cells, likely due to its disruption of Cdc50 mediated lipid flippase function.

### Lysine residues are involved in key interactions in analogous flippase-Cdc50 structures.

We sought to examine the interaction of our new lead compound, AW9-Ma with the P4-ATPase. To do so, we examined the homologous structure of an ATPase-PS-Cdc50a complex in several cryo-EM structures. We first identified the complementary region in the human P4-ATPase structures. This was an attempt to find the binding site where the Cdc50a exoplasmic loop interacts with the P4-ATPase. Sequence alignment of the fungal Cdc50 shows that only a portion of the amino acids are conserved between Homo sapiens and C. neoformans. Nonetheless, this region has several similarities, and can be recognized as related to the source of the original AS15 sequence from C. neoformans (Fig. S3 in the supplemental material). We therefore deduce that the interactions of this region of the H. sapiens Cdc50a with the H. sapiens P4-ATPase are analogous to the interactions of AS15 or AW9 with the C. neoformans P4-ATPase. We analyzed several existing Cryo-EM derived structures of Homo sapiens flippase ATP11C, which was found in human RBCs as it interacts with Cdc50 to translocate PS molecules (24). We focused in on the analogous region for our peptide AW9-Ma. The structures were screened for any interactions between the protein and the “peptide,” the analogous region on Cdc50a which were within 5 Å. Three common interactions were found, lysine-glutamine, tryptophan-valine, and lysine-glycine/alanine (Table 5).

**TABLE 5:** Interactions between the defined region of the Cdc50 loop and the ATP11C protein^*a*^

Protein	Protein – Cdc50a	Interaction
7bsp	**K** 988 – G 203: 3.3 Å **V** 989 – W 206: 2.1 Å **Q** 1051 – K 213: 2.2 Å	H-bond, K side chain – G carbonyl H-bond, V carbonyl – W side chain N-H H-bond, Q carbonyl – K side chain N-H
7bsq	**K** 988 – A 205: 2.4 Å **V** 989 – W 206: 2.3 Å **Q** 1051 – K 213: 1.8 Å	H-bond, K side chain – A carbonyl H-bond, V carbonyl – W side chain N-H H-bond, Q carbonyl – K side chain N-H
7bss	**K** 988 – A 205: 2.6 Å **V** 989 – W 206: 2.1 Å **Q** 1051 – K 213: 2.4 Å	H-bond, K side chain – A carbonyl H-bond, V carbonyl – W side chain N-H H-bond, Q carbonyl – K side chain N-H
7bsu	**K** 988 – G 203: 2.8 Å **V** 989 – W 206: 2.4 Å **Q** 1051 – K 213: 2.7 Å	H-bond, K side chain NH – G carbonyl H-bond, V carbonyl – W side chain N-H H-bond, Q carbonyl – K side chain N-H
7bsv	**K** 988 – A 205: 2.5 Å **V** 989 – W 206: 2.3 Å **Q** 1051 – K 213: 1.9 Å	H-bond, K side chain – A carbonyl H-bond, V carbonyl – W side chain N-H H-bond, Q carbonyl – K side chain N-H
7bsw	**K** 988 – A 205: 2.9 Å **V** 989 – W 206: 2.2 Å **Q** 1051 – K 213: 2.0 Å	H-bond, K side chain – A carbonyl H-bond, V carbonyl – W side chain N-H H-bond, Q carbonyl – K side chain N-H

a Key interactions were identified as those where the ATP11C protein was within 5 Å of the homologous Cdc50 peptide loop region. Here the “protein” amino acid is indicated in bold, and the “peptide” amino acid is shaded.

**FIG 7:**
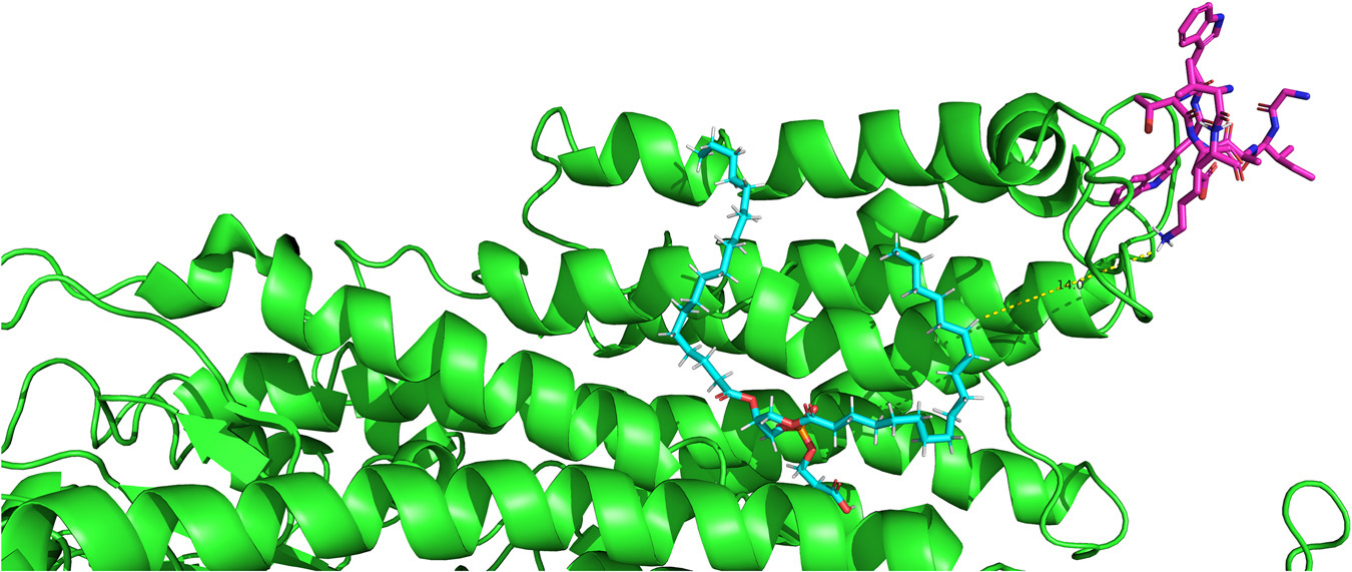
Structure of homologous lipid flippase *Homo sapiens* ATP11C-CDC50a loop (7BSU) interaction. The 14.0 Å distance between Cdc50 loop region and phosphatidylserine in the flippase protein with green (flippase), purple (Cdc50), blue (phosphatidylserine) are highlighted.

These key interactions are, therefore, most likely analogous to the interactions between AW9 and the P4-ATPase as these are the similar residues in both. The fatty acid tail of the AW9-Ma peptide likely binds closely to this region as well. We hypothesized that the most likely binding pocket for the AW9-Ma would be located where the PS molecule exists in these structures. The closest of these interactions is modeled below in PyMol (Fig. 7). Interestingly, the distance between the end of the closest amino acid and the hydrogen of the fatty acid tail is 14 Å. This is roughly the length of a myristic acid. Thus, the lipid tail is within this distance for interacting with the hydrophobic pocket of P4-ATPase where the PS is located. This observation is supported by the previously described data showing that fatty acid tails shorter than 14 carbons deplete the peptide activity.

### Modifying peptide AW9-Ma to replace lysine for alanine removes activity.

From the analysis of analogous structures, we hypothesized the lysine residues of the AW9-Ma peptide were crucial for antifungal activity. To address this possibility, three further peptides were created by changing either one or both lysine residues to alanine. These peptides had modifications at residue number 5 lysine (K6A-Ma), residue number 6 lysine (K6A-Ma), and both (K5A;K6A-Ma). Our method was similar to the common molecular biology alanine scan technique but using solid phase peptide synthesis rather than mutagenesis (25). The change from lysine to a nonpolar alanine has the potential to disrupt hydrogen bonding between the flippase and the peptide. Indeed, among these peptides, each of them exhibited a higher MIC value toward the H99 wild-type peptide than the parent peptide (Table 6). These data suggest that the lysine residues are key for interaction of the peptides with the flippase and are thus partly responsible for their antifungal activity.

**TABLE 6:** The MIC values of lysine→alanine mutant peptides against C. neoformans^*a*^

Peptide	Sequence	MIC (μg/mL)
K5A-Ma	WGGIAKNYA	>128
K6A-Ma	WGGIKANYA	>128
K5A;K6A-Ma	WGGIAANYA	>128

a The sequences of each peptide are shown along with their corresponding MIC values. In all cases, the peptides were inactive against C. neoformans.

**FIG 8:**
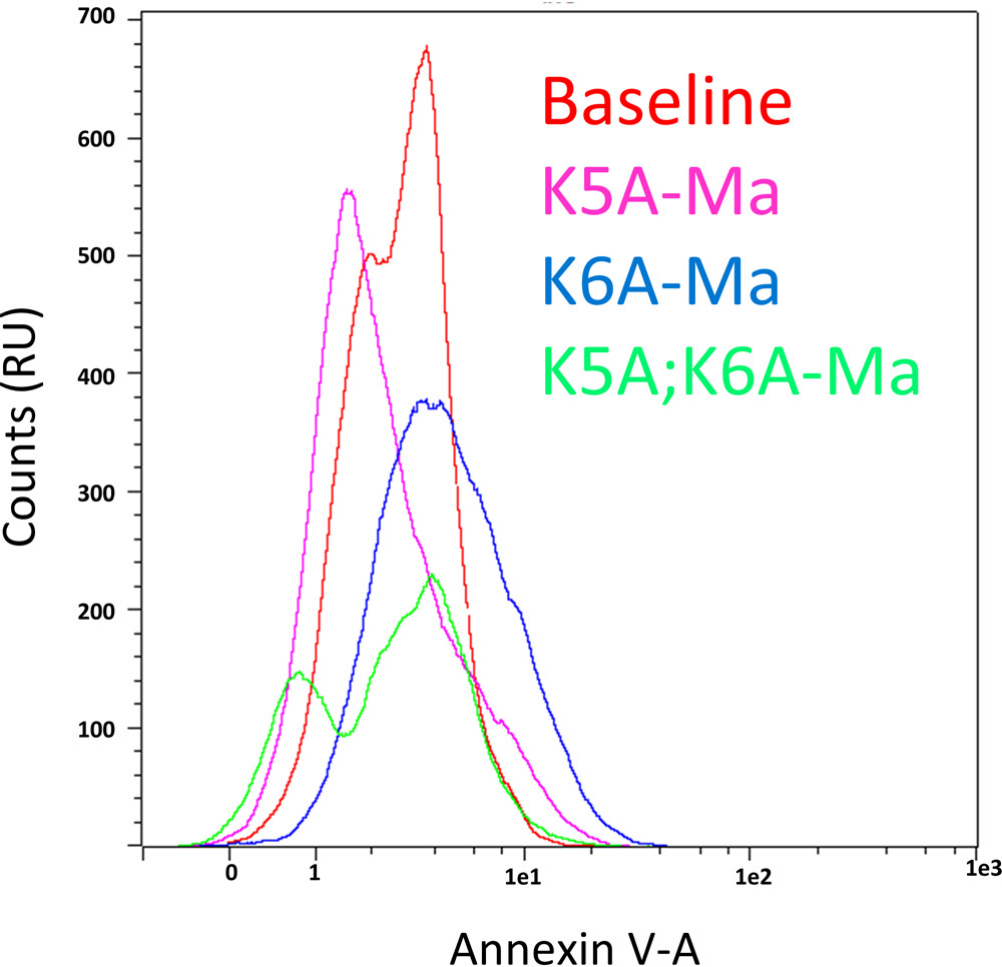
Histogram of Annexin V Signal Counts in C. neoformans treated with peptides. Cells were treated using the same methods as for the active peptides. Relative counts (RU) of annexin V signal were recorded. Signal for the peptides is relatively close to baseline.

In addition to having different MIC values, the peptides also have a different effect on the accumulation of exofacial PS. For the peptide K5A-Ma the percentage of cells in the gated region of interest was 5.6 ± 0.1%, which was significantly lower than the AW9-Ma peptide (Student's *t* test, *P*-value < 0.05). Interestingly, the K6A-Ma showed a more modest decrease in the positive cell population of 17.9 ± 2.1%, though this was still a significant decrease from the AW9-Ma peptide (Student's *t* test *P*-value < 0.05). Most interestingly, the double alanine substitution peptide K5A;K6A-Ma caused only 1.3 ± 0.9% of cells to be in this range. (Fig. 8) This value was not even statistically significantly different than the control, untreated cells (Student's *t* test *P*-value > 0.1). Retention of one lysine residue provides some activity, with the residue number 6 lysine apparently being more important. It is clear that replacing both lysine residues depletes peptide activity.

### Conclusion.

We have developed peptides based on the Cdc50a loop region that have antifungal activity against C. neoformans. The most successful peptide was labeled AW9-Ma and was derived from nine residues in Cdc50 with a myristic acid tail. Furthermore, when used in combination with caspofungin, the FIC of caspopfungin drop to 4 μg/mL. We note that this is the same value as the previously reported MIC for the *cdc50Δ* mutant strain against caspofungin. This peptide not only successfully inhibited the growth of H99 C. neoformans, but it also caused significant changes in the properties of the cell membrane. We detected the peptides accumulation on the membrane and its co-localization with the flippase P4-ATPase Apt1. The accumulation of exofacial PS can have a detrimental effect on the stability of the membrane (26). One such effect is an increased susceptibility of peptide-treated cells to SDS similar to the *cdc50Δ* phenotype. The fungal cells treated with AW9-Ma showed increased exofacial phosphatidylserine as monitored by an AF350-annexin V-PI assay. Together, these results suggest that AW9-Ma binds to the flippase and promotes exofacial PS on the cell membrane.

**FIG 9:**
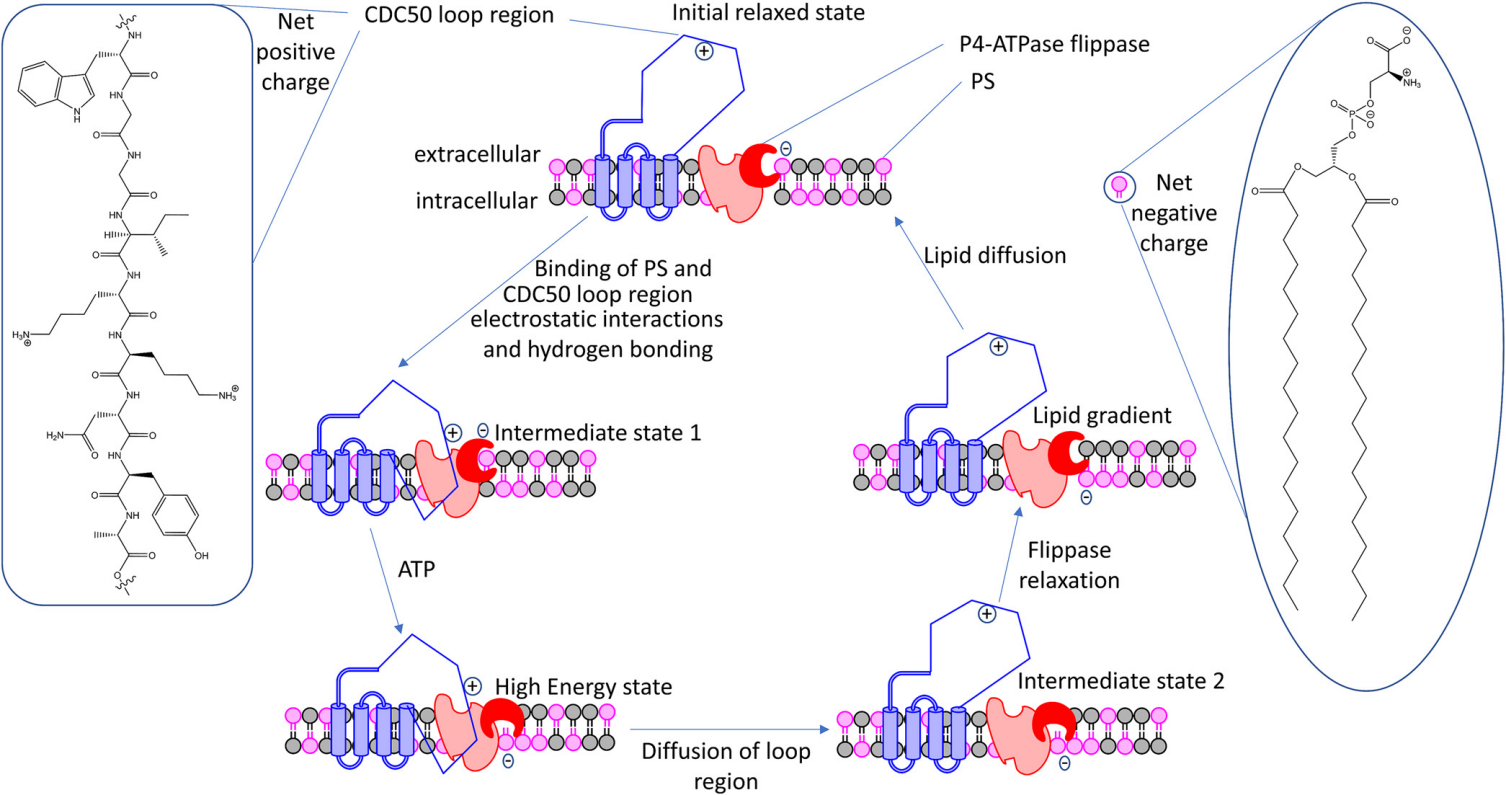
Possible mechanism of action of Cdc50 and P4-ATPase in C. neoformans. Fungal loop region interacts with the peptide in a manner to facilitate the turnover of a region in the peptide (dark red) which allows for the movement of lipids from exofacial to internal.

**FIG 10:**
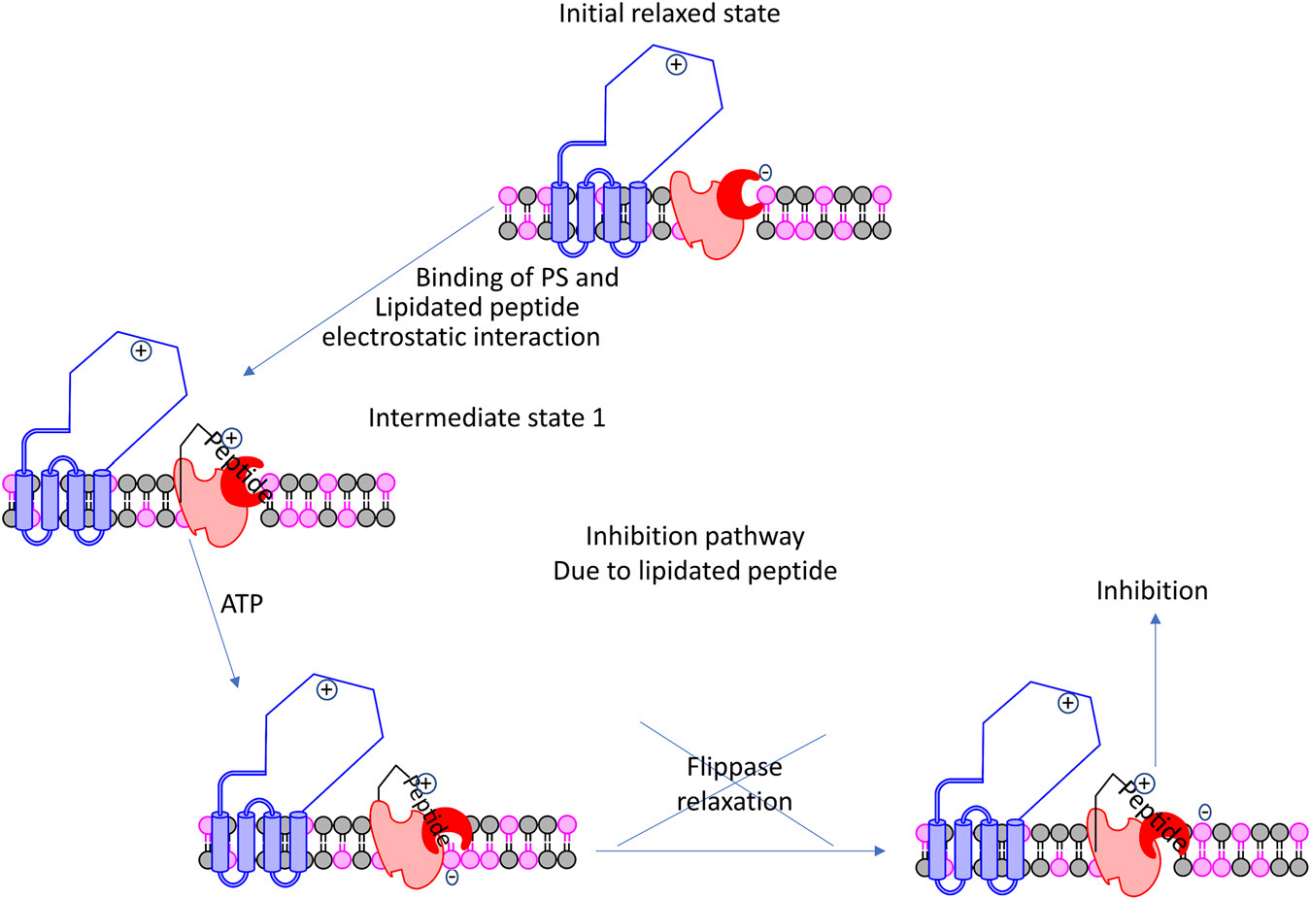
Proposed mechanism of action of antifungal peptides. Peptides bind to P4-ATPase through hydrogen bonding interactions with charged residues, this prevents the turnover of the region responsible for lipid flipping. This model of activity is consistent with the peripheral pathway model of flippase activity.

Taking these results into consideration we propose a mechanism of peptide activity. Opening of channel region (red) is required for shuttling of lipids from one side of the membrane to the other (Fig. 9) (27). Normal P4-ATPase function is regulated by diffusion of the Cdc50 loop region out of the binding pocket in the P4-ATPase-Cdc50 complex. This is required for the flippase to relax back to an initial state (28, 29). In the absence of the peptide, the Cdc50 loop region binds to complex through specific hydrogen bonds and electrostatic interactions. In the presence of a molecule such as AW9-Ma, these bonds are replaced by interactions with the peptide (Fig. 10). This is supported by our data showing that truncated peptides lacking the lysine residues as well as Lys→Ala modified AW9-Ma all have decreased activity. The enzyme will eventually become locked in a peptide-bound conformation, unable to relax back to the initial state. Thus, further lipid flipping is prevented.

Future work is required to further validate the precise mechanism of the peptide action. Specifically, an alanine scan of Cdc50 *in vivo* could address whether Lys→Ala mutants exhibit a similar phenotype to *cdc50Δ*. Modifications to the peptides can be implemented in the future to tune the specificity of the peptide to fungal membranes over mammalian membranes. Possible modifications include post translational modifications, like side chain glycosylation, cyclization, substitution with amino acid derivatives, or unnatural -D amino acids. Along these lines, future peptide modifications should attempt to tune the hydrophobic moment of the peptide to fungal membranes with different types of branched and unsaturated fatty acid tails, or fatty acid tail derivatives like Beta-hydroxymyristic acid. Further complications exist in the fact that the importance of 1,3-β-D glucan for C. neoformans survival is debated (30). Overall, however, AW9-Ma is a promising lead candidate with a plausible activity that could serve as a useful molecule to improve C. neoformans susceptibility to caspofungin.

## MATERIALS AND METHODS

### Materials.

Piperidine and acetic anhydride were supplied by Alfa Aesar (Haverhill, MA). Caspofungin was supplied by AM Beed (Arlington Heights, IL). Sodium hydroxide was supplied by Avantor (Philipsburg, NJ). Yeast peptone dextrose media was supplied by Beckton Dickinson (Franklin Lakes, NJ). Formic acid and Sabouraud dextrose agar were supplied by Merck (Kenilworth NJ). Wang resin and fluorenylmethoxycarbonly (Fmoc) protected amino acids were supplied by Novabiochem (Burlington, MA). O-(1H-6-Chlorobenzotriazole-1-yl)-1,1,3,3-tetramethyluronium hexafluorophosphate (HCTU) was supplied by Peptides International (Louisville, KY). Bio-Rad Assay Reagents were supplied by Bio-Rad (Richmond, CA). Sulfuric Acid was supplied by Pharmco (Brookfield CT). Alexafluor350 and Alexafluor350-labeled Annexin V were supplied by Thermo Fisher Scientific (Waltham, MA). Dimethyl formaldehyde (DMF), Dimethyl sulfoxide (DMSO) and Acetonitrile (ACN) were supplied by VWR (Radnor, PA). Human red blood cells (RBCs) were supplied by Interstate Blood Bank (Philadelphia, PA). All other materials and reagents were supplied by Sigma-Aldrich (St. Louis, MO).

### Western blot analyses.

Total proteins from animal tissues (Bone Marrow, Heart and Lung), Cryptococcus neoformans cells (wild-type strain H99, *fbp1Δ* mutant strain and *cdc50Δ* mutant strain), and mouse macrophage cell line J774 were extracted. Protein extraction was performed as previously described (31). Animal tissue cells, yeast cells and macrophage cells were rapidly frozen in a dry ice/ethanol bath, resuspended in lysis buffer (50 mM Tris-HCl [pH 7.5], 1% (wt/vol) sodium deoxycholate, 5 mM sodium pyrophosphate, 10 nM sodium orthovanadate, 50 mM NaF, 0.1% (wt/vol) SDS, and 1% (vol/vol) Triton X-100) containing a cocktail of protease inhibitors (0.5 mM phenylmethylsulfonyl fluoride, 1 μg of pepstatin mL^−1^, 1 mM benzamidine, and 0.001% aprotinin) with 1–1.2 g of acid-washed glass beads and disrupted using a FastPrep instrument (FastPrep-24 5G, MP Biomedicals, CA). Protein concentrations were determined by Bio-Rad Protein Assay reagent. Proteins were loaded into a 10% Tris-glycine gel, and separated proteins were further transferred to Immuno-blot PVDF membrane (Bio-Rad) and incubated overnight at 4°C with a primary rabbit Cdc50 specific antibody (GenScript, Piscataway, NJ) and with a secondary anti-rabbit IgG horseradish peroxidase-conjugated antibody. The blot was developed using the ECL Western Blotting Detection System (Amersham Bioscience, Piscataway, NJ). Subsequently, the blot was stripped and further used for detection of Actin, with a mouse beta-actin monoclonal antibody (GenScript) as a loading control.

### Peptide synthesis.

Microwave assisted (CEM Discover), solid phase peptide synthesis (SPPS) was used to synthesize the peptides. Each peptide was made using standard FMOC, tBu protection scheme at 0.05 to 0.1 mmol scale. The first amino acid coupling was repeated 4 times to ensure quantitative labeling to wang resin 100–200 mesh before proceeding to the first deprotection. All other amino acids were coupled once. For the first two groups of peptides, coupling ingredient molar ratios were 5:5:10:1, amino acids:HCTU:DIEA:peptide. Excess 20% piperidine in DMF was used as the deprotection solution. Subsequent peptides were synthesized with molar ratios of 3:3:6:1 respectively. Fatty acid tails (FATs) were conjugated to the N terminus of the peptide using their respective anhydrides C2-C14, and DIEA, 1:1:1, FAT: DIEA: peptide. Palmitic C16 FAT was coupled using palmitic acid, HCTU, and DIEA, 1:1:3:1, FAT: HCTU: DIEA: Peptide. All fatty acid tails were double coupled to ensure maximum labeling. Peptides were cleaved in 6 mL tubes for 1 h using 95%:2.5%:2.5% trifluoroacetic acid (TFA):Triethylsilane (TES):H_2_O (v:v:v). Resulting solution was collected quantitatively in a 50 mL falcon tube. A steady stream of N_2_ gas was used to evaporate the liquid. The remaining plug of material was diluted to 25 mL in 10% acetic acid in water. The sample was vortexed and sonicated for 30 min before freezing in the –80°C freezer overnight. The frozen peptide solutions were lyophilized and stored for further purification.

### Purification and liquid chromatography.

A Varian Prep Star chromatographic system (Palo Alto, CA) was used to purify the peptides. Samples were diluted to 15 mL in 30% acetonitrile in water with 0.1% formic acid (FA) buffer. The mixture was vortexed and heated with a heat gun until sample was mostly dissolved. The mixture was clarified by filtering through 0.45 μm filter tip. Clarified filtrate (5-8 mL) was loaded into a 10 mL injection loop. Preparatory column used was Phenomenex, Luna 10 C18(2), 250 × 21.20 mm, 10 μ. A 5 mL per minute flow rate was used for a gradient of 5-95%ACN. The 220 nm and 280 nm channels were used to detect the sample via dual channel detector. Fractions were collected in 50 mL falcon tubes and were switched by hand. Fractions were selected, combined, and worked up based on the mass spectra and analytical HPLC traces. Combined volumes were reduced to 20-30mL on a roto-evaporator and acidified to 10% acetic acid V/V. Solutions were vortexed and sonicated for 10 min before freezing overnight in the –80°C freezer. Subsequent solids were lyophilized to dry powder and characterized. Results are provided in Table S4 in the supplemental material.

Analytical HPLC was performed using a Shimadzu Nexera - i LC-2040C 3D plus device. The column used was a Waters XBridge Shield RP 18, 3.5 μ, 3.0x150mm. Peptides were prepared at 1 mg/mL in 40% ACN with 0.1% FA and filtered through 0.22 μm filter. Experiments were performed with a flow rate of 1 mL/min. A mobile phase gradient of 5–95% ACN (H_2_O with 0.1% FA and ACN with 0.1% FA). Data was recorded using a PDA spectrometer with a spectral window of 200 – 456 nm, a resolution of 256, and a frequency of 30 Hz.

### Mass characterization.

Peptide masses were verified using several different methods. Peptides were characterized using an ESI-MSD single quad device, HP Series 1100 (Palo Alto, CA). This was done using 10 μL direct injections with a flow rate of 0.5 mL/min. In these experiments, the mobile phase was 90% MeOH in water with 0.1% FA. Peptides were prepared at 1 mg/mL in 40% ACN with 0.1% FA and filtered through 0.22 μm filter. Some peptides were further analyzed using a triple quad device, Perkin Elmer QSight 225MD (Waltham, MA). Clarified filtrate from single quad sample prep was diluted by a factor of 1000 in ACN with 0.1% FA to 1 μg/mL for direct infusion at 30 μL/min. Further analysis by MALDI-TOF was provided by the Mass Spectroscopy Lab (University of Illinois, Urbana, IL). Results table is provided in Table S5 in the supplemental material.

### Cryptococcal minimum inhibition concentration (MIC) assay.

Our assays followed standard CLSI protocol for broth microdilution assays using yeasts (32). Microbes were passaged in YPD media at 37°C with mixing for 2 days before harvesting with an inoculation loop to prepare monocultures on Sabouraud Dextrose Agar (SDA) plates. Monocultures on SDA plates were incubated for 2 days at 37°C before harvesting cells for microdilution assays. RPMI media (100 μL) was charged to each of the 96 wells in a 96-well plate, with an additional 100 μL in the first column. Peptide-DMSO solution (4 μL) at 12.8 mg/mL was charged to each of the wells in the first column. The 100 μL was serially diluted by a factor of 2 from the first column to the 11th column. Microbe inoculant was then prepared. An inoculation loop was used to transfer 1–3 colonies of cells to 1 mL phosphate buffer solution (PBS). The PBS and microbe solution was made uniform by vortex mixing. Cell concentration was computed by comparing the absorbance at 530 nm to 1x McFarland standard solution and multiplying the resulting ratio by 10^7^ cell/mL conversion factor. Plate inoculant was prepared by diluting the PBS microbe solution to 5000 cell/mL, in RPMI media. A 100 μL aliquot of cells was transferred to each of the wells on the plate other than column 11 for the no growth control. The operating volume was 200 μL, with 500 CFU per well for a final concentration of 2.5*10^3^ cells/mL. Inoculated plates were incubated for 48 h at 37°C and read at 24 and 48 h (33).

### Checkerboard assay.

Microbes were prepared in the same manner as the MIC assay. The first set of drug dilutions were prepared in a similar manner as the MIC assay except for 4 μL peptide-DMSO stock solution was charged to the wells instead of 2 μL and drugs were serially diluted to column 8 instead of 11. 100 μL of RPMI media was added to the first 8 wells in the first row. A 2 μL aliquot of Peptide-DMSO stock solution at 12.8 mg/mL was added to each of the 8 wells in the first row. The 100 μL of media with drug was serially diluted from the first row to the final row. Columns 9 and 10 were used as MIC control wells for the two drugs. Column 11 was the no growth control, and 12 was the no drug control. The plate was inoculated with microbes in the same manner as the MIC assay. Again, 200 μL was the operating volume, 500CFU per well, 2.5*10^3^ cell/mL. Plates were incubated for 48 h at 37°C and read thereafter. The Fractional Inhibitory Concentration Index were calculated in Equation 1:

$$\mathrm{FIC}\;\mathrm{Index}=\frac{\mathrm{FI}C_{A}}{\mathrm{MI}C_{A}}+\frac{\mathrm{FI}C_{B}}{\mathrm{MI}C_{B}}$$

Where the MIC_A_ and MIC_B_ are the MIC values of drugs A and B alone and FIC_A_ and FIC_B_ are the Fractional Inhibitory concentrations of drugs A and B in the presence of each other, respectively (34).

### Hemolysis assay.

Hemolysis assays were preformed using human red blood cells (RBCs), which were provided by Interstate Blood Bank (Philadelphia, PA) and stored immediately at 4°C upon arrival. A description of the method development for this assay is further provided in Supporting Information. Free heme was extracted from stored RBCs washing in PBS with gentle centrifugation at 1000 RPM for 5 min. Washed cells resuspended to 4 mL in PBS and counted using a hemocytometer. Hemolysis assay plates were prepared by charging 50 μL PBS to each well of an 8 × 8 grid on the 96-well plate, and an additional 50 μL aliquot PBS to the first 8 wells in row A. A 2 μL aliquot of peptide-DMSO stock solution at 12.8 mg/mL was transferred to 4 of the wells in row A. A second peptide-DMSO stock solution was transferred in the remaining 4 wells in row A. A 50 μL aliquot of solution was then serially diluted by a factor of 2 down the plate from row A to row H. A 50 μL aliquot of PBS was charged to 8 additional wells on the plate to account for the positive and negative controls. Red blood cells were adjusted to 5*10^8^ cells/mL and 50 μL was added to each of the wells. Operating conditions were 100 μL and 2.5*10^8^cell/mL. The positive control used as 2 μL of 1% Triton X-100. The plate was incubated at 37°C for 1 h. The resulting plate was spun down at 1000 RPM for 5 min. 20 μL supernatant was transferred to 80 μL PBS in a fresh 96-well plate for analysis (operating volume 100 μL.) A Spectra Max 340 plate reader (Molecular Devices, Sunnyvale, CA) was used to measure the absorbance of the analysis plate at 410 nm. The scans were run in triplicate. Averages and standard deviations between scans and replicates were computed. The negative control was subtracted from all the data points. The resulting corrected data was normalized by the corrected positive control.

### AF350-annexin V flow cytometry assay.

Stock microbes were defrosted from the –80°C freezer and were passed twice for 48 h each in YPD media. The YPD media was removed by spinning down and the supernatant was decanted. Pelletized cells were resuspended in 1 mL PBS media and washed for 5 min with incubation at 25°C and 250 RPM. Washed cells were spun down again and suspended in 1 mL of PBS solution. cells were diluted 20-fold into 1 mL fungal working solution in an eppendorf. Cell concentration was quantified via comparison to 1X McFarlain’s standard on a plate reader using the absorbance value at 530 nm. An aliquot of 1*10^6^ cells in 100 μL was created (1*10^7^cell/mL). A 100 μL aliquot of peptide working solution at 0.256 mg/mL was added to the cell suspension for final operating parameters of 200 μL, 0.128 mg/mL peptide, and 5*10^6^cell/mL. The cells were incubated for 20 min at 25°C and 250RPM. Baseline and experimental samples were prepared in triplicate. Cells were stained using an operating concentration of 0.001 mg/mL Propidium Iodine (PI) and a 40-fold dilution of Alexa Fluor 350 labeled annexin V as per manufactures instructions. After staining the cells, samples were spun down and supernatant decanted. Cells were then fixed in 4% formaldehyde for 5 min. Fixed cells were spun down and supernatant decanted and resuspended in 400 μL 1% formaldehyde for flowcytometry. Samples were collected at a slow flow rate on a MACS Quant 10 device (Miltenyi Biotech, Auburn, CA) with 50,000 events collected per injection.

### SDS and plate spotting assay.

To test cell integrity, C. neoformans strains from 3 mL YPD overnight cultures were washed, resuspended, and serially diluted (1:10) in dH2O and spotted (5 μl) on YPD agar plates or YPD agar plates containing 0.03% SDS and incubated for 2 days at 30°C and 37°C. To test the effect of peptide AW9-Ma on cell phenotype. Of each strain, yeast cells were inoculated into liquid YPD with 128 μg/mL or 64 μg/mL peptide and co-incubated for 1h at 30°C. And then, 10-fold serial dilutions were prepared, and 5 μl of each was spotted on YPD and YPD agar plates containing 0.03% SDS. The results were photographed after a 48 h of incubation. Their growth was determined by serial dilutions and the fungal colonies were assessed by CFU numbers.
