# Supplementary material for: Development of Antifungal Peptides against Cryptococcus neoformans; Leveraging Knowledge about the cdc50Δ Mutant Susceptibility for Lead Compound Development
Source: Microbiol Spectr. 2022 Apr 4;10(2):e00439-22. doi: 10.1128/spectrum.00439-22 (PMC9045296; doi:10.1128/spectrum.00439-22)
Supplement: SUPPLEMENTAL FILE 1 — Supplemental material. Download SPECTRUM00439-22_Supp_1_seq4.pdf, PDF file, 0.2 MB [file spectrum00439-22_supp_1_seq4.pdf]

**Sequence**	***MIC ug/mL***		***FIC ug/mL***				**FIC Index**	
	H_{99}	*ΔCDC_{50}*	*H_{99}*		*ΔCDC_{50}*		*H_{99}*	*ΔCDC_{50}*
			Caspofungin	*Peptide*	Caspofungin	*Peptide*		
Caspofungin	16	4	-	-	-	-	-	-
QY15	>128	>128	8	0.5	1	0.5	0.5-1	0.5-1
QY15-Ma	>128	128	8	0.5	1	0.5	0.5-1	0.25
AS15	>128	128	8	0.5	1	0.5	0.5-1	0.25
AS15-Ma	>128	8	8	0.5	0.25	0.25	0.5-1	0.094
AS15-Aa	>128	>128	-	-	-	-	-	-
AS15-Ha	>128	>128	-	-	-	-	-	-
AS15-Da	>128	>128	-	-	-	-	-	-
AW9-Ma	64	2	4	16	0.125	0.125	0.5	0.094
KS9-Ma	>128	>128	-	-	-	-	-	-
GS9-Ma	>128	>128	-	-	-	-	-	-
AS15-Pa	>128	128	8	0.5	2	2	0.5-1	0.53
AW9-Pa	>128	8	8	0.5	2	1	0.5-1	0.75

S1. Table comprising all values of MIC, FIC, and FIC Index determined throughout the paper against wild-type and mutant strains.

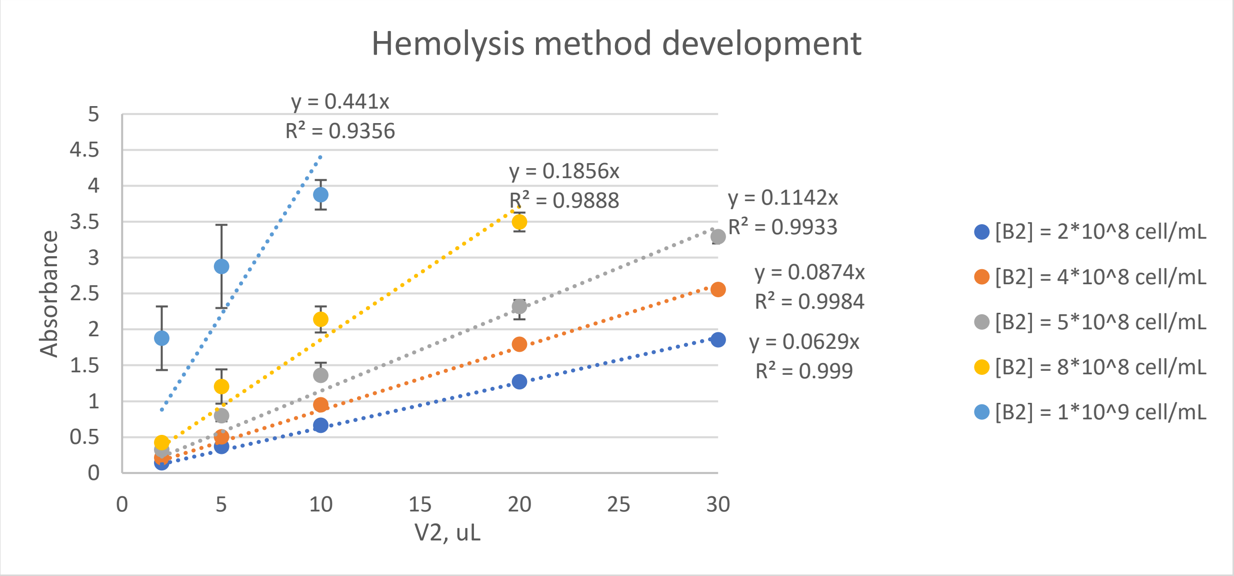

S2. Figure showing calibration of the hemolysis experiment

```
HsCdc50A        GIFVTSNNIREIEIDYTGTEPSSPCNKCLSPDVTPCF-----------------------
CnCdc50         -----SGKVTTITLDYTECDVDAPTDGSYQAMPNSAYQYDLATSSSVSESSIASPTWTFS
                     *.::  * :***  :  .:* :  .  ..   ...:

HsCdc50A        ------------CTINFTLEKSFEGNVFMYYGLSNFYQNHRRYVKSRDDSQLNGDSSALL
CnCdc50         NDSSREVGETARCEIEFEVPYDLGPGLFLYYKLTNYYQNHRRYSSSFDATQLIGDSRSLS
                            * *:* :   .:   .:*:** *:*:******* .* * :** *** :*

HsCdc50A        N-PSKECEPYRRNEDKPIAPCGAIANSMFNDTLELFLI---GNDSYPIPIALKKKGIAWW
CnCdc50         QINGGNCKPITSRDGKPYYPCGLIANSLFNDTFPSVVLLNPTNGAQNQTYNFSESGIAW-
                :   . :*:*    .:.**  *** ****:****:   .::    *.:    .   :.:.****

HsCdc50A        TDKNVKFRNPPGGDNLEERFKGTTKPVNWLKPVYMLDSDPDN------NGFI--------
CnCdc50         -----------GG--IKKNYAST---LTYISPSDVL--PPPNWALKYPNGYVDGFPNLRE
                           **  :::.: .*    :.::.*  :*    * *       **::

HsCdc50A        NEDFIVWMRTAALPTFRKLYRLIERKSDLHPTLPAGRYSLNVTYNYPVHYFDGRKRMILS
CnCdc50         DEHFQVWMRVAALPTFRKLWARNDGE-----IMSQGRYRIVANMNYPVKQFSGTKSIVIS
                :* * ****.*********:    : :       :. *** : .. ****: *.* * :::*
```

S3. Alignment of *Homo sapiens* CDC50 protein with *Cryptococcus neoformans* CDC50. The boxed region shows the location of the original peptide sequences.

Peptide	Amount mg (Crude)	Yield % (Crude)	Amount mg (Purified)	Yield % (Purified)	Purity (%)
AS15	176.23	111.5	123.98	78.43	99.2
AS15-MA	199.5	111.4	120.61	67.34	97.5
QY15	191.52	102.6	68.86	36.9	96.4
QY15-MA	186.19	89.67	133.68	64.38	96
AS15-AC	190.91	117.6	162.15	99.9	99.0
AS15-HEX	160.78	95.76	91.82	54.7	99.3
AS15-DA	179.14	103.3	132.13	76.2	98.4
AW9-MA	129.83	104.1	77.64	62.3	87.6
KS9-MA	107.14	97.54	72.85	66.3	81.1
GS9-MA	105.90	98.76	36.72	34.2	70.5
AS15-Pa	178.10	97.9	105.43	59.7	76
AW9-Pa	134.51	105	58.24	46.5	93.7

S4. Yield and purity of peptides before and after HPLC purification.

Peptide	Expected Mass (Da)	Calculated m/z			Crude		Purified	
		+1	+2	+3	Observed m/z	Comment	Observed m/z	Comment
AS15	1580.76	1581.77	791.39	527.92	790.5	+2	790.4	+2
AS15-Ma	1791.13	1792.13	896.57	598.04	895.5	+2	895.5	+2
QY15	1865.98	1866.99	934.0	622.99	932.9	+2	932.9	+2
QY15-Ma	2076.34	2077.35	1039.18	693.11	1038.3	+2	1038.4	+2
AS15-Ac	1622.80	1623.80	812.4	541.93	1623.1, 812.5	+1, +2	812.3	+2
AS15-Ha	1678.91	1679.91	840.455	560.63	1679, 839.45	+1, +2	840.5	+2
AS15-Da	1733.94	1734	867.97	578.98	867.55	+2	869.6	+2
AW9-Ma	1246.56	1247.56	624.28	416.52	1246.95, 623.6	+1, +2	1247.7; 624.0	+1, +2
KS9-Ma	1098.40	1099.40	550.2	367.13	1099, 550.6	+1, +2	1098.7, 551.0	+1, +2
GS9-Ma	1072.27	1073.27	537.135	358.42	N/A	N/A	1095.6	+1, Na adduct
AS15-PA	1819.18	1820.18	910.59	607.39	909.2	+2	910.0	+2
AW9-PA	1274.62	1275.62	638.31	425.87	1274.3, 637.7	+1, +2	1275.6; 637.8	+1, +2

S5. Mass Spectroscopy results for peptide predicted monoisotopic mass
